# Multi-Level Features Extraction for Discontinuous Target Tracking in Remote Sensing Image Monitoring

**DOI:** 10.3390/s19224855

**Published:** 2019-11-07

**Authors:** Bin Zhou, Xuemei Duan, Dongjun Ye, Wei Wei, Marcin Woźniak, Dawid Połap, Robertas Damaševičius

**Affiliations:** 1 School of Sciences, Southwest Petroleum University, Chengdu 610500, China; duan_xuem@163.com (X.D.); dongjunye@yeah.net (D.Y.); 2 Institute of Artificial Intelligence, Southwest Petroleum University, Chengdu 610500, China; 3 Research Center of Mathematical Mechanics, Southwest Petroleum University, Chengdu 610500, China; 4 School of Computer Science and Engineering, Xi’an University of Technology, Xi’an 710048, China; 5 Institute of Mathematics, Silesian University of Technology, 44-100 Gliwice, Poland; marcin.wozniak@polsl.pl (M.W.); Dawid.Polap@polsl.pl (D.P.); Robertas.Damasevicius@polsl.pl (R.D.)

**Keywords:** feature, tracking, WMSNs, matching, weight, multi-level

## Abstract

Many techniques have been developed for computer vision in the past years. Features extraction and matching are the basis of many high-level applications. In this paper, we propose a multi-level features extraction for discontinuous target tracking in remote sensing image monitoring. The features of the reference image are pre-extracted at different levels. The first-level features are used to roughly check the candidate targets and other levels are used for refined matching. With Gaussian weight function introduced, the support of matching features is accumulated to make a final decision. Adaptive neighborhood and principal component analysis are used to improve the description of the feature. Experimental results verify the efficiency and accuracy of the proposed method.

## 1. Introduction

Remote sensing technology and wireless multimedia sensor networks (WMSNs) have been widely used in various fields of the national economy and are able to collect lots of data such as video and audio streams, still images, and scalar sensor data from the environment. It has been one of the most interesting research fields in the past few years [1,2,3].

Remote sensing monitoring is the remote observation of the characteristics or phenomena of the target through monitoring devices such as infrared detectors, multimedia sensors, and some other electronic or optical instruments. It means monitoring and analyzing a target/phenomenon without directly contacting the target/phenomenon when collecting information. Remote sensing technology can be used to quickly locate the ecological environmental pollution sources or other interested targets [4,5].

WMSNs are often composed of many wirelessly interconnected devices such as low-cost hardware CMOS cameras, microphones, and other sensor nodes with computational and wireless sensing capabilities; they can help to complete varies of tasks in remote sensing [2,6,7].

With the advances in wireless and electronic technologies, a variety of intelligent systems based on WMSNs have been developed for target tracking, behavioral analysis, identification, traffic surveillance, healthcare monitoring, environment monitoring, and so on [8,9]. Many techniques are presented to address these tasks based on video sequence analysis [10,11,12], and they are advantageous in ideal urban scenes with abundant computational ability.

A supervised learning framework is presented to generate compact and bit-scalable hashing codes directly from raw images [13]. Then, the deep convolutional neural network is utilized to train the model with the image features and hash functions simultaneously optimized. [10] proposes a novel network named part-based convolutional baseline (PCB) based on a convolutional descriptor consisting of several part-level features and a refined part pooling method for person retrieval. [14] presents a new object tracking approach for surveillance applications developed using a big data model based on graphs and a multilevel fusion. With enough quantity of pose-rich samples generated from the original image and skeleton samples, a novel unsupervised pose augmentation cross-view scheme is proposed for person re-identification [12]. In [15], an improved method is developed to detect and track multiple heads by considering them as rigid body parts for real-time video surveillance. The appearance model of human heads is updated according to the fusion of color histogram and oriented gradients. Acoustic and image hybrid wireless multimedia sensors’ networks are introduced to trajectory prediction for target tracking [16].

However, there are still some tasks must be completed in special scenes with limited computational ability or power support, such as wildlife monitoring and tracking [1,17]. Remote sensing image monitoring based on an optimized convolutional neural network model is introduced to the conservation of rare wild animals [1]. A hierarchical wireless sensor network is installed in Doñana National Park to collect information about animals’ behaviors. The placed intelligent devices contain a neural network implementation to classify the animals’ behavior [17]. A novel energy-efficient object detection based on the image transmission approach is proposed for wireless multimedia sensor networks [18].

Similar contents in different images can be found by analyzing the pixel values and their potential features, named features’ detection and image matching [19,20,21]. Traditional features’ detection approaches are started with Harris’s corner detection and Forstner’s work on the fast operator for precise location of distinct points [22,23]. In addition, various methods and algorithms are then developed with different comprehensive understanding of the features [21,24,25].

Distinctive invariant features can be extracted to perform reliable matching between different views of an object or scene [20], and this named scale-invariant feature transform technology has been widely applied in lots of computer vision tasks. The principal component analysis is introduced to describe the feature points and the dimension-reduced descriptor can hold some advantageous in robustness or computation [26]. By relying on integral images for image convolutions and using a Hessian matrix-based measure, a novel scale-and rotation-invariant interest point detector and descriptor (namely Speeded Up Robust Features, SURF) is presented and it approximates or even outperforms previously proposed methods respected to robustness and computation [27]. With the maximum similarity measure defined in terms of geometric and photometric properties of regions, a hierarchical image matching based on a tree matching problem is presented to identify the largest similar part [28]. A practical method is proposed to establish dense correspondences between two images with similar content, but possibly different 3D scenes [29].

In the traditional scenes, with the sufficient support of computation ability and power, enough image sequences are easy to be captured and convenient to be analyzed. Many previous methods are presented based on this ideal situation. However, sometimes, the targets move from one camera to another, and very short video or a few images can be captured for the limited environment condition or device condition. In addition, the relation among the videos or images may be loose or not so continuous. In this case, feature detection should be implemented more efficiently to finish the task with less computation and power.

In recent years, convolution neural networks and machine learning have been rapidly developed in many fields. Large scale databases are often applied to training various deep and structural models. Though a considerable efficiency achieved in the scenes covered by these data, it may be also mean that it is more difficult to adapt some scenes with unexpected data. Furthermore, it is not easy to collect enough data in any scenes. Thus, it is still necessary to address some tasks via interpretable methods with some mathematical base. In this paper, a novel multi-level features extraction is approached for discontinuous target tracking in remote sensing image monitoring. Multi-level features of the reference image are pre-extracted. The rough features are used to exclude the obvious error targets. In addition, the refined features are used to compare with the rest candidate target. Adaptive neighborhood and the principal component analysis (PCA) are used to describe the feature. The weighted support of matching features will be accumulated based on a Gaussian function to make the final decision.

The rest of this paper is organized as follows. The related fundamentals of remote sensing monitoring, target tracking, and features extraction are prepared in Section 2. The multi-level features extraction and discontinuous targets tracking are proposed in Section 3. Several experiments are implemented in Section 4 to verify the accuracy and efficiency of the proposed method.

## 2. Fundamentals

### 2.1. Remote Sensing and Wireless Multimedia Sensor Networks

There are many techniques, such as photography, infrared scanning, correlation spectroscopy, lidar detection, and unmanned aerial vehicles, that can be used to achieve remote sensing monitoring [30,31]. Remote sensing cameras can be remotely monitored by installing them on a flying device or on a satellite to capture targets on the ground, vegetation, and plant emissions. The principle of remote sensing technology is that the reflection characteristics of electromagnetic waves are often not the same due to different objects or phenomena, and photographs of different colors or tones can be obtained by photosensitive recording of the photosensitive film. In some cases, the surveillance area can be deployed with a wireless distributed sensor network consisting of a set of multimedia sensor nodes, so-called wireless multimedia sensor networks. These nodes are connected or connected to the main gateways using a wireless communication protocol.

**Figure 1:**
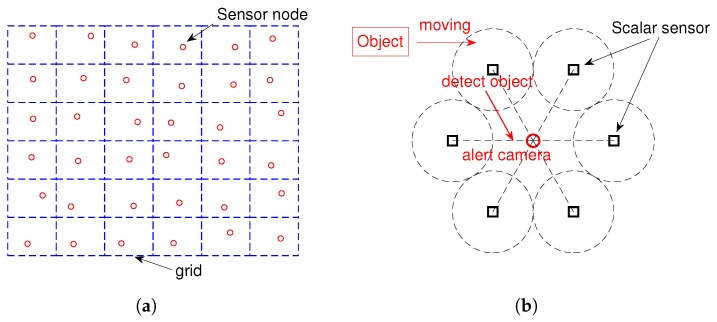
Topology and workflow of wireless multimedia sensor networks: (**a**) topology of the networks; (**b**) workflow of the networks.

Suppose there are *N* sensor nodes deployed in a square surveillance zone that is divided into n×n grids. The grid approach is commonly used to monitor the entire area without leaving gaps between the sensor nodes [32]. Each sensor node is fixed on a position $\left(x_{s},y_{s}\right)$, and there are several scalar sensors (such as seismic and acoustic) that are deployed around for detecting moving targets and awaken the camera sensor. As an object enters the grid area, it will be first detected by a scalar sensor. Then, the camera sensor will be awakened and try to take a short video or some images according to the position of alerting the scalar sensor. Figure 1 shows the topology and workflow of wireless multimedia sensor networks.

Our goal is to track a moving target using such sensor networks composed of camera sensor nodes and scalar sensors.

**Figure 2:**
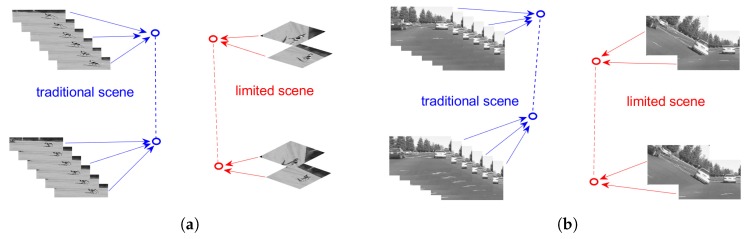
Illustration of a traditional and limited scenes: (**a**) Dog case; (**b**) Panda case. In both cases, a number of obtained frames can be seen.

Different from traditional urban scenes, the computation ability and power support are limited. The cameras only work as they are awakened and very short videos or very few images can be captured for recognition and tracking. The multimedia data may be linked to several objects moving, and it means that the videos or images are not continuous in the spatial or time. It will be more difficult to recognize and track the target with such a discontinuous data. Figure 2 shows the difference between traditional scenes and limited scenes.

In the traditional scene, with the powerful support of energy and computation ability, enough long video and lots of image sequences can be easily collected for the latter computing. Many popular deep learning methods can be applied to complete the tasks. However, in a limited scene, there are only a few short videos and images available.

### 2.2. Multi-Cam Tracking and Re-Identification

The research about disjoint cameras is started with Huang and Russell’s work on Bayesian formulation. They use the formulation to estimate the posterior of predicting the appearance of objects in one camera given evidence observed in other camera views. Multiple spatial-temporal features such as color, vehicle length, height and width, velocity, and time of observation are all included in the appearance model [33,34].

The term “person re-identification” is first proposed by Zajdel, Zivkovic, and Krose [35] in the research about multi-camera tracking.

They aim to recognize a person when it leaves the field of view and re-enters later. A dynamic Bayesian network is defined to encode the probabilistic relationship between the labels and features (color and spatial-temporal cues).

After then, many technologies are developed to address this problem such as independence of re-ID (image-based), video-based re-ID, deep learning for re-ID, end-to-end image-based re-ID, and so on [34,36,37].

### 2.3. Feature Extraction

Feature extraction is one of the most important techniques in computer vision and many high-level applications must be implemented on it [21,26]. Earlier approaches are started with the detection of corner points or distinct points [23]. Based on the local auto-correlation function, Harris proposed a combined corner and edge detector to cater for image regions containing texture and isolated features [22].

An important milestone of feature extraction is the presentation of scale-invariant feature transform (SIFT) [20]. This local image features is proposed to develop an object recognition system, and it is invariant to image scaling, translation, and rotation, and partially invariant to illumination changes and affine or 3D projection.

To reduce the 128-dimensional feature descriptor, principle component analysis is introduced to normalized gradient patch instead of using smoothed weighted histograms [26].

In addition, various methods and algorithms are then developed with different views to features [24,25,38]. Speeded Up Robust Features (SURF) is a novel interest point descriptor with scale-and rotation-invariance based on image convolution and Hessian matrix-based measure [27]. Some other methods are also presented by different principles such as geometric and photometric properties, dense correspondence, human visual system, dedicated sampling, and so on [29,39].

## 3. Proposed Method

### 3.1. Optimal Selection to the Principle Components

By optimizing a closed-world toy model, Gheissari et al. [40] addresses person re-identification based on single-image. *G* is assumed to be a gallery composed of *m* images, denoted as ${\left\{g_{i}\right\}}_{i=1}^{m}$. It means there are *m* different identities, 1,2,⋯,m. Given a probe image collected by WMSNs, its identity can be determined by

(1) $$\begin{array}{c} i^{*}={\mathrm{argmax}}_{i}\;sim\left(q,g_{i}\right), \end{array}$$

where $i^{*}$ means the decision and sim(·,·) means the similarity function. In general, the similarity can be computed based on the image features such as SIFT, SURF, etc.

**Figure 3:**
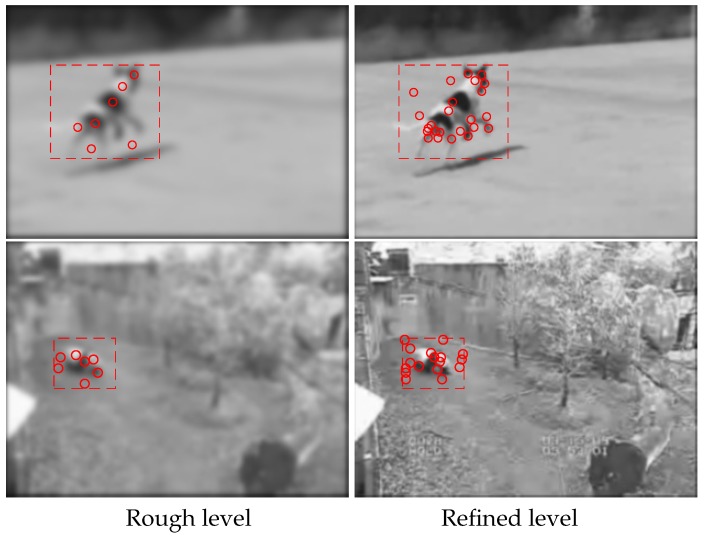
Multi-level features points of reference images, where features are described as a red circle, and a detected object is surrounded by a rectangle. In the first column, the rough features are marked, and, in the second one, refined ones.

Multi-level features points of each image in the reference gallery are pre-extracted based on the classical SIFT as shown in Figure 3—the first column shows the rough feature points and the second one shows refined feature points.

Assume that $p_{i,j}^{\left(k\right)},j=1,2,\cdots ,n_{i}$ denotes the kth level features points of image $g_{i}$. In this paper, k=1 means rough level and k=2 means refined level. Then, each b×b local area centered a rough level point is reshaping to a row vector after necessary rotated normalization. All the vectors are arranged to a matrix denoted by $H^{\left(k\right)}$ and apply principle component analysis on it. The main advantages of using principal component analysis are that the method is performed without supervision, so there is no need to have any information about classes during size reduction. As a result, the method indicates the dominant patterns in the analyzed sets.

Let $H^{\left(k\right)}\left[i,j\right]$ denote the reshaped row vector of the normalized local area centered $p_{i,j}$. To well separate the multi-targets, a set of components to describe the feature points should be determined by

(2) $$\begin{array}{c} \begin{array}{c} \max_{\delta }\sum_{i_{1}\neq i_{2}}\sum_{j_{1},j_{2}}\left|\right|H^{\left(k\right)}\left[i_{1},j_{1}\right]diag\left(\delta \right)V-H^{\left(k\right)}\left[i_{2},j_{2}\right]diag\left(\delta \right)V{\left|\right|}^{2}\\ s.t.\\ \delta =[\delta_{1},\delta_{2},\cdots ,\delta_{b^{2}}],\\ \delta_{s}\in \left\{0,1\right\},s=1,2,\cdots ,b^{2}. \end{array} \end{array}$$

Here, *V* denotes the components’ matrix. The binary vector δ means selection to the components. Maximizing the objective function means to maximize the distance between two different targets.

### 3.2. Image Matching via Refined Feature Describing

A probe image can be checked by model (1) with a proper similarity threshold set. If passed, it will be further matching via refined features.

Scale-invariant feature transform (SIFT) is a common descriptor widely applied in many computer vision problems such as image matching, object recognition, and so on. Though some classical improvements have been approached in recent years, it still is one of the most representative techniques to well describe the image features. Based on the feature points computed previously, the traditional SIFT can be implemented as three main steps:

Step 1. Determine candidate key-points via peak selection in the difference of Gaussian space;
Step 2. key-point checking and orientation assignment;
Step 3. Eight direction statistics and key-point describing.

To make it work well in these limited WMSN scenes, we introduce adaptive neighborhoods to key-point checking and PCA to orientation assignment. This paper presents a novel frame for multi-targets tracking in limited WMSNs, and the feature extraction method can be directly replaced if necessary. For better performance of feature describing, some other techniques can also be introduced to these steps.

### 3.3. Evaluation of the Matching Results

Suppose that there are some feature points $pos_{i,j}^{\left(k\right)}\left(j=1,2,\cdots ,m_{i}\right)$ in the probe image *q* found to be matching the feature points $p_{i,j}^{\left(k\right)}$ in a reference image $g_{i}$. The aggregation of $pos_{i,j}^{\left(k\right)}$ and $p_{i,j}^{\left(k\right)}$ can be measured independently to make a binary decision on the target tracking.

It is natural to introduce the Gaussian weight function to measure the aggregation of $p_{i,j}^{\left(k\right)}$ in the reference image and regard it as similarity:

(3) $$\begin{array}{c} \begin{array}{c} sim\left(q,g_{i}\right)=\frac{1}{m_{i}}\sum_{j=1}^{m_{i}}\exp\left(-\frac{r_{j}^{2}}{2\sigma^{2}}\right),\;r_{j}=p_{i,j}^{\left(k\right)}-\frac{1}{m_{i}}\sum_{j=1}^{m_{i}}p_{i,j}^{\left(k\right)}, \end{array} \end{array}$$

where σ denotes a distance scale factor and $r_{j}$ means the distance from $p_{i,j}^{\left(k\right)}$ to their center.

However, the aggregation of $pos_{i,j}^{\left(k\right)}$ in the probe image *q* is ignored and the above measurement can be improved as

(4) $$\begin{array}{c} \begin{array}{c} sim\left(q,g_{i}\right)=\frac{1}{m_{i}}\sum_{j=1}^{m_{i}}\exp\left(-\frac{r_{j}^{2}}{2\sigma^{2}}\right)+\frac{1}{m_{i}}\sum_{j=1}^{m_{i}}\exp\left(-\frac{s_{j}^{2}}{2\sigma^{2}}\right),\\ r_{j}=p_{i,j}^{\left(k\right)}-\frac{1}{m_{i}}\sum_{j=1}^{m_{i}}p_{i,j}^{\left(k\right)},\;s_{j}=pos_{i,j}^{\left(k\right)}-\frac{1}{m_{i}}\sum_{j=1}^{m_{i}}pos_{i,j}^{\left(k\right)}, \end{array} \end{array}$$

Then, the similarity can be easily applied to make a binary decision on target tracking.

(5) $$\begin{array}{c} \mathrm{IsIdentifying}\left(q,g_{i}\right)=\left\{\begin{array}{cc} 1,\; & sim\left(q,g_{i}\right)>\rho_{0},\\ 0,\; & sim\left(q,g_{i}\right)\leq \rho_{0}. \end{array}\right. \end{array}$$

Though in a discontinuous tracking scene, it is still assumed that a few images could help to determine the moving area. Then, there is a probability of whether a feature belongs to the target. It is similar to a matching. Several intensive matching means more probability of target identification. Equation (5) is introduced to determine the identification based on the concentration of the matching.

For more accurate computing, some other techniques can be introduced to improve the similarity such as adaptive weight function, distance metric learning, and so on.

## 4. Experiments

**Figure 4:**
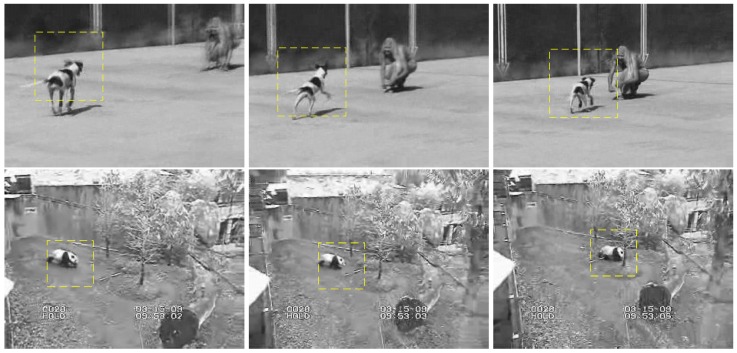
Sample images with marked moving objects in yellow rectangles.

In limited scenes, very few images can be collected to match test and target tracking. However, we still assume that there are two images of each moving object that can be captured by a camera each time. The object in the probe image can be located by the difference of the convolution with the Gaussian kernel. Figure 4 shows the object location computed from the two probe images. For well matching results, the local areas have been extended to include most feature points of the target and nearby surroundings.

We present several experiments to evaluate the performance of the proposed method. The first and second are implemented to explore the recognition ability of our method to discontinuous probe images. Each pair of probe images are selected from a continuous sequence of images and the interval between them is more than five frames. The third and last are used to explore the recognition ability of the proposed method to multi-targets tracking. The experiment data are downloaded from a Visual Tracker Benchmark (v1.0) [41]. All the experiments are completed under Windows 7 system with Matlab R2017b. The related parameters are set as follows. The features matching was determined traditionally by Euclidean distance and the ratio between the shortest one and the second shortest one. The ratio threshold is set to be 0.6 in this paper. The distance scale factor σ in Equation (4) is set to be $\frac{d}{2}$ and *d* means the local image area size. The similarity threshold $\rho_{0}$ is set to be 0.6.

Features matching test 1. Four pairs of images of a dog are selected from the Dog image sequence in OTB-100. Frames 1, 11, 18, and 31 are assumed to be reference images and frames 6, 16, 23, and 36 are regarded as probe images.

**Figure 5:**
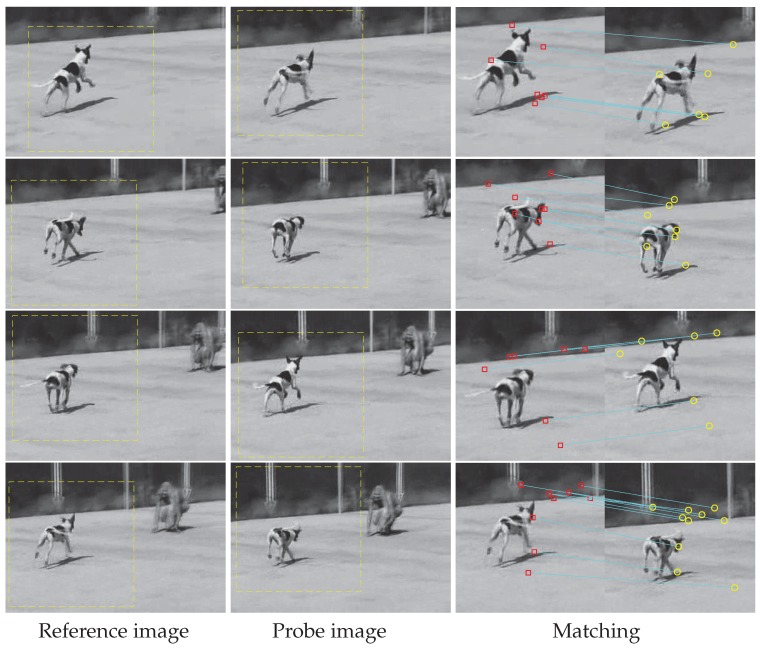
First feature matching test. In the first two columns, moving objects are marked, and, in the last column, there are marked key-points (as red squares and yellow circles for better visualization) with an indication of their position on two different frames with a blue line.

As shown in Figure 5, the left column means reference images with moving detection, the middle means probe images with moving detection, and the right column is the matching results. The rows mean different image pairs and the detected moving areas are 200×200. It can be found that there are 7–9 matches in each pair. Some are related to the dog self and others are related to the surroundings. It is interesting that several means the correspondence of shadows.

**Figure 6:**
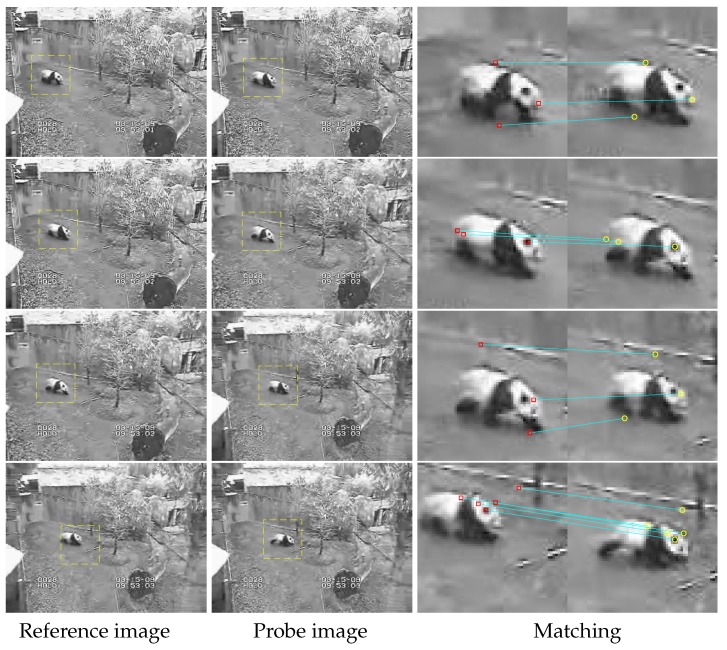
Second feature matching test. In the first two columns, moving objects are marked, and, in the last column, there are marked key-points (as red squares and yellow circles) with an indication of their position on two different frames with a blue line.

Features matching test 2. Four pair images of a panda (shown in Figure 6) are selected from the Panda image sequence in OTB-50. Frames 1, 11, 23, and 31 are assumed to be reference images and frames 10, 22, 34, and 42 are regarded as probe images.

The columns from left to right mean reference images with moving detection, probe images with moving detection, and the matching results. The rows correspond to different image pairs.

It can be found that there are a few matches in each pair compared to experiment 1 because of the smaller local image area (61×61). Most of the matches are related to the panda self and very few related to the surroundings.

Target tracking test 1. Frame 1 is selected from the Dog image sequence and supposed to be a reference image. Frames 9, 16, 23, and 30 are regarded as probe images captured by different cameras.

**Figure 7:**
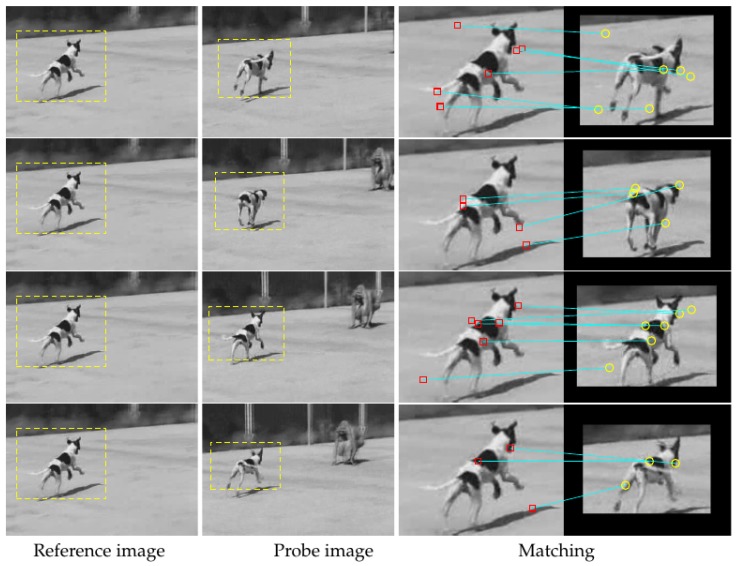
First target tracking test. In the first two columns, moving objects are marked, and, in the last column, there are marked key-points (as red squares and yellow circles) with an indication of their position on two different frames with a blue line.

As shown in Figure 7, the images in the first column are the same as frame 1, which is regarded as a reference image. Probe images with moving detection are shown in the second column. Matching results are shown in the last column.

The detected moving areas are often in different size and they are adaptively computed in the tracking. It can be found that there are about five matches in each pair.

**Figure 8:**
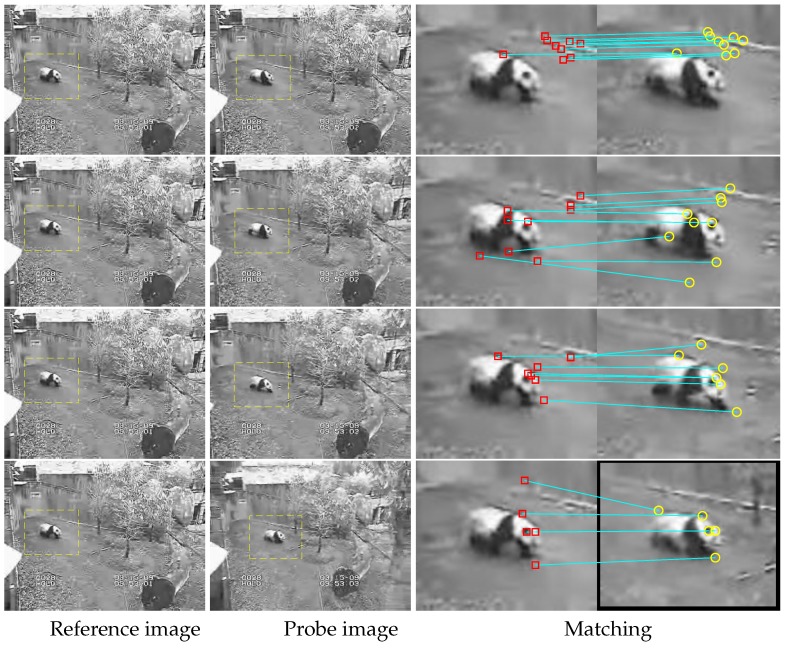
Second target tracking test. In the first two columns, moving objects are marked, and, in the last column, there are marked key-points (as red squares and yellow circles) with an indication of their position on two different frames with a blue line.

Target tracking test 2. Frame 1 is selected from the Panda image sequence and supposed to be a reference image. Similar to the above test, we select frames 9, 16, 23, and 30 to be probe images for tests, as shown in Figure 8. Different probe images with moving detection are shown in the middle column. Matching results are shown in the last column.

**Figure 9:**
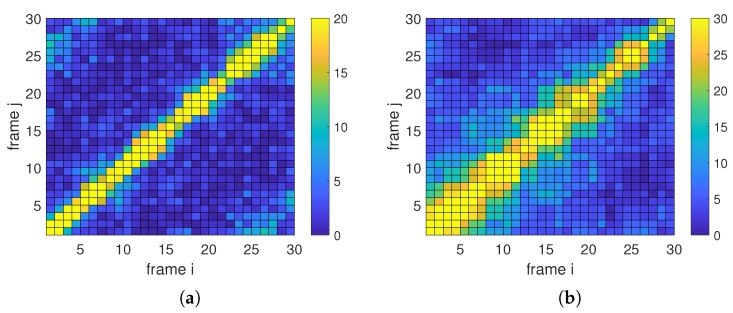
Matching relations of discontinuous frames: (**a**) matching relation of Dog frames; (**b**) matching relation of Panda frames.

More details about the matching relation of frame 1 to frame 30 are shown in Figure 9. The color value at position (i,j) means the matching number of frame *i* and frame *j*. It is found that more matching can be captured between a pair of close images, and there is a lot of matching that can be found in most of the image pairs. However, there is not yet matching that can be found in a few image pairs. Thus, the features describing and matching still should be improved for serious discontinuous targets tracking, although the proposed method has provided a solution to this problem in some sense.

**Remote sensing image matching test**. There are two image sequences (sequence A and sequence B) from a scene. The camera views and scales are much different from each other. We try to explore the tracking ability of the proposed method from three points.

**Figure 10:**
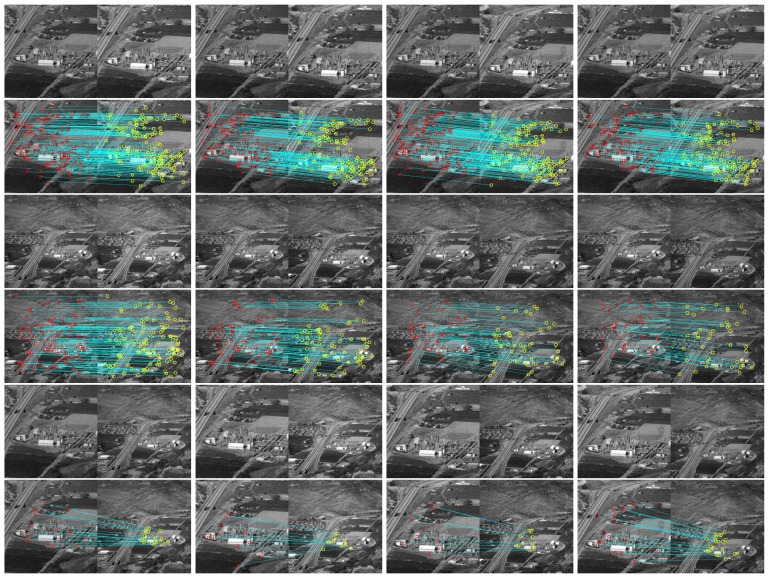
Remote sensing image matching test—the original frames are shown in the odd rows, and, the even ones, frames with the found key-points (marked as yellow and red points) and their locations as a blue line.

(1) Suppose frame 1 of sequence A to be a reference image and then frames 7, 13, 19, and 25 are selected to test the tracking ability. The moving area is set to be the whole image. Original image pairs are shown in the first row of Figure 10 and matching results are shown in the second row.

(2) Frame 1 of sequence B is supposed to be a reference image and then frames 3, 5, 7, and 9 are selected to test the tracking ability. Original image pairs and matching results are shown in the third and fourth row.

(3) Two images are selected independently from sequence A and sequence B to generate an image pair for discontinuous targets tracking ability test. Original image pairs and matching results are shown in the fifth and sixth row.

It can be found from the results that more matching can be captured in (1) than (2) because of the short shooting distance. Though a significant scale difference between sequence A and sequence B can be found, there are still considerable matching that can be captured in each image pair crossed the wide-scale gap.

In these discontinuous targets tracking scenes, sequence analysis-based methods or learning-based methods are difficult to get to work well because of insufficient data. The methods based on single-image re-identification require considerable computation for features detection on each probe image and matching them to the reference images. However, sometimes, it is not necessary to introduce refined computing at first. Furthermore, little guarantee can be achieved for distinguishing different targets because of the independent features describing. Compared to the traditional methods, a two-stage procedure is introduced to reduce some unnecessary computation. In addition, the optimal set of components based on PCA is applied to well distinguish the features from different targets. These contribute to the effectiveness of the proposed method.

## 5. Conclusions

In this paper, a multi-level features extraction is presented for discontinuous target tracking in remote sensing image monitoring. The features of reference images are extracted at different levels in advance. The rough-level features are used to discard the error target and refined-levels are used to target matching. Proper neighborhood can be set adaptively and principal component analysis is used to improve the descriptor. The weighted support of matching features can be accumulated to make the final decision. Experimental results verify the efficiency and accuracy of the proposed method.
